# Efficacy of articaine infiltration versus lidocaine inferior alveolar nerve block for pulpotomy in mandibular primary second molars: A randomized clinical trial

**DOI:** 10.15171/joddd.2018.015

**Published:** 2018-06-20

**Authors:** Sara Ghadimi, Mahdi Shahrabi, Zahra Khosravi, Rooholah Behroozi

**Affiliations:** ^1^Laser Research Center of Dentistry, Department of Pediatric Dentistry, School of Dentistry, Tehran University of Medical Sciences, Tehran, Iran; ^2^Department of Pediatric Dentistry, School of Dentistry, Tehran University of Medical Sciences, Tehran, Iran; ^3^Department of Pediatric Dentistry, School of Dentistry, Hamadan University of Medical Sciences, Hamadan, Iran; ^4^Department of Endodontics, School of Dentistry, Hamadan University of Medical Sciences, Hamadan, Iran

**Keywords:** Anesthesia, articaine, lidocaine, pulpotomy

## Abstract

***Background:*** Successful anesthesia is a major concern in during pulpotomy treatment. The aim of this study was to compare the anesthetic efficacy of inferior alveolar nerve block using 2% lidocaine and buccal infiltration using 4% articaine for pulpotomy of mandibular primary second molars.

***Methods:*** This randomized cross-over clinical trial was performed in 23 children (five to eight-year-old) from July through November 2016, referred to the Department of Pediatric Dentistry, Tehran University of Medical Sciences who needed pulpotomy treatment in both mandibular primary second molars. The Patients’ feeling during injection and their behavior during pulpotomy and post-treatment complications were registered. Wilcoxon Signed Ranks test was used for analyzing the data. A significant level of differences was taken as p≤ 0.05.

***Results:*** Patients’ feeling during injection and post-treatment complications did not significantly differ between two groups (p>0.05). Patients’ behavior during pulpotomy was significantly better in articaine group (p=0.004).

*** Conclusion:*** Articaine buccal infiltration can be used successfully in pulpotomy of mandibular primary second molars. Iranian Registry of Clinical Trial: (IRCT2015042321484N2).

## Introduction

Pain control is mandatory to reduce anxiety during dental treatments, particularly in children.^1^ IANB is the preferred technique for achieving pulpal anesthesia during treatment of mandibular primary molars.^2^ Clinical studies have reported the failure of IANB as high as 44‒84%, necessitating supplementary injections.^3-5^ Two percent lidocaine, the most commonly used anesthetic agent in dentistry, generally in the IANB technique.^1^ Buccal infiltration (BI) using 2% lidocaine is not as effective as the IANB for achieving profound anesthesia in mandibular molars, due to the low penetration of anesthetic solutions through the buccal cortical plate.^6-8^ The prolonged soft tissue anesthesia frequently associated with IANB could result in self-inflicted trauma such as biting of lip/cheek.^1^ Four percent articaine was introduced to the clinical practice in 1976 and has been claimed to be more effective than lidocaine. Articaine is an amide anesthetic solution with an additional ester group; it also contains a thiophene ring which enhances its lipid solubility and allows the solution to easily penetrate into the tissues.^9^ In human studies, 4% articaine has been reported to be more successful than 2% lidocaine when used as BI in adult subjects.^10-12^

Various trials have compared the efficacy of 4% articaine with 2% lidocaine in permanent teeth. However, the majority of these studies have been performed on adult subjects and data regarding their effectiveness in children are proportionately sparse. Therefore, the current study was conducted to compare the efficacy of 4% articaine BI with 2% lidocaine IANB for pulpotomy in mandibular primary second molars.

## Methods

This randomized crossover clinical trial was conducted on 23 children (5‒8 years of age) referred to the Department of Pediatric Dentistry, Tehran University of Medical Sciences, who needed pulpotomy treatment in both mandibular primary second molars. A power calculation consisting of α=0.05 and β=0.2, standard deviation=1.04 and minimum significant difference=0.84 was carried out using Minitab 17 statistical software (Minitab Inc, State College, PA, USA) and indicated that a sample size of 21 subjects in each group would be sufficient.

### Inclusion criteria

- Cooperative behavior for dental treatment under local analgesia (class 3 or 4 in Frankel scale).^6^
- No history of allergic reactions to local anesthetic solutions or sulfites.
- No medical conditions contraindicating the use of local anesthetics containing epinephrine.
- No evidence of soft tissue infection/inflammation near the area of injection.
- Not taking any medications that potentially interfere with pain assessment within 24 hours before the treatment.
- No difficulties in communication.

**Figure 1:**
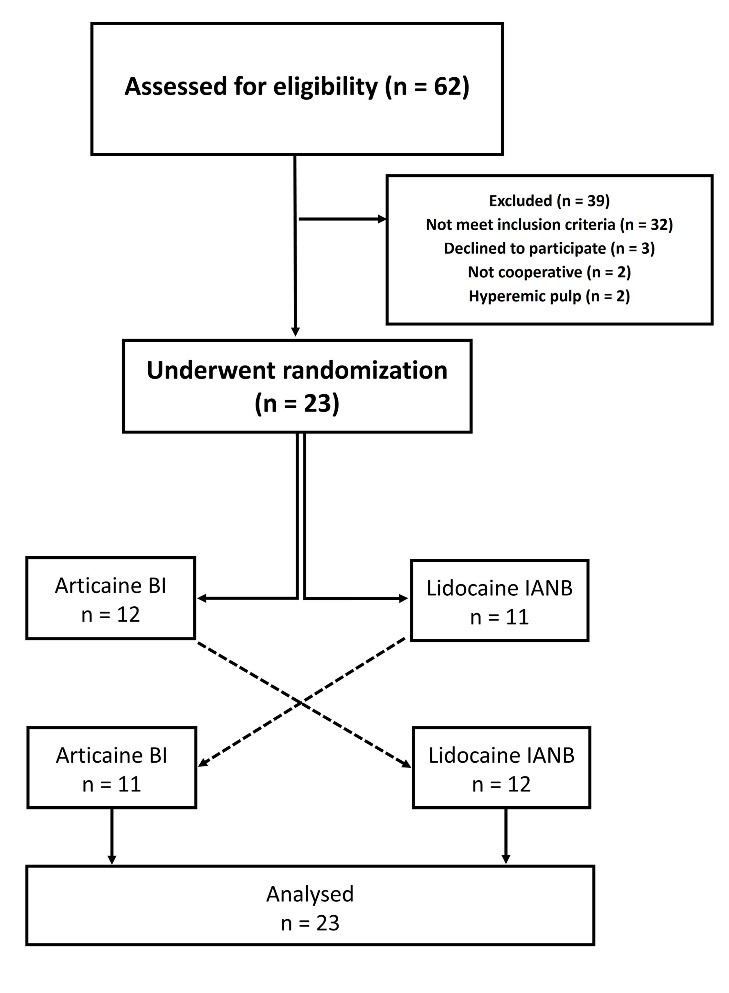
Study flow diagram.

**Figure 2:**
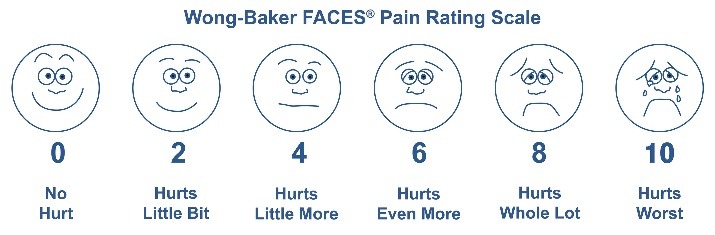
Wong-Baker FACES pain rating scale.

The study protocol was approved by the Ethics Committee in Medical Research of Tehran University of Medical Sciences (IR.TUMS.REC.1394.610). The trial was registered in the Iranian Registry of Clinical Trials (http://www.irct.ir) (IRCT2015042321484N2). Written informed consent was taken from all the participants. The patients were allocated to two groups using random numbers table in Excel 2013 (Microsoft Corporation, WA, USA). To ensure allocation concealment, the numbers were kept in opaque and sealed envelopes, which were opened by an assistant who was blinded to the scheme of the study. In the first appointment, 11 subjects received 1.8 mL of 4% articaine with 1:100,000 epinephrine (Articaine 100, DFL, Rio de Janeiro, Brazil) as BI and 12 subjects received 1.8 mL of 2% lidocaine with 1:80,000 epinephrine (Persocaine, DarouPakhsh Co, Tehran, Iran) as IANB (Figure 1). In the second appointment, with at least a one-week interval from the first appointment, the other solution was administered. Prior to the injection, topical anesthetic gel (Benzotop, DFL, Rio de Janeiro, Brazil) was applied with a cotton roll for one minute. Local anesthetic solutions were delivered using a standard aspirating syringe with a 30-gauge needle (Septoject, Septodont Inc, New Castle, DE, USA). Anesthetic solutions were injected at a rate of approximately 1 mL/min. All the injections were performed by a pedodontist who was unaware of the anesthetic solution and the scheme of the study. Immediately after the injections and verbal instructions, the each child was asked to select the facial expression that best represented his/her feeling of discomfort according to Wong-Baker FACES pain rating scale (WBFPRS, Figure 2).^13^ All the pulpotomy treatments were performed by the senior author who was blinded to the injection techniques and the type of anesthetic solution used. During the pulp excavation, the modified behavioral pain scale (MBPS) suggested by Taddio et al^14^ (Table 1) was used for objective evaluation of the patients’ behavior; this scale includes the facial expression, movements and crying. A trained staff member blinded to the type of anesthetic solutions completed the scale for all the subjects during the pulpotomy procedure. The presence of any adverse events including infection, headache, oral/tooth pain, self-inflicted soft tissue traumas such as biting of the lip/cheek was established by two follow-up telephone calls 24 hours and one week after the treatment. Wilcoxon signed ranks test was used for analysis of data. A significant level of differences was taken as P≤0.05.

**Table 1:** Modified Behavioral Pain Scale suggested by Taddio et al13

**Parameter**	** Finding **	** Points **
** Facial expression **	Definite positive expression (smiling)	0
	Neutral expression	1
	Slightly negative expression (grimace)	2
	Definite negative expression (furrowed eye brows; eyes closed tightly)	3
** Cry **	Laughing or giggling	0
	Not crying	1
	Moaning quietly; vocalizing gentle or whimpering cry	2
	Full crying or sobbing	3
	Full crying more than baseline cry (scored only if child was crying at baseline)	4
** Movements **	Usual movements and activity	0
	Resting and relaxed	0
	Partial movement (squirming, arching limb; tensing, clenching)	2
	Attempt to avoid pain by withdrawing the limb where the puncture is done	2
	Agitation with complex/generalized movements involving the head, torso or other limbs	3
** Rigidity **	3

Modified behavioral pain scale = SUM (points for all 3 parameters); 0, minimum score; 10, maximum score

## Results

Twenty-three children, 13 boys and 10 girls, 5‒8 years of age (average: 6.25 years) participated in this randomized, double-blind clinical trial. The weight of subjects was 16.5‒29.0 kg (average: 20.76 kg). The mean of pain felted during injection according to WBFPRS was 3.30±2.93 in the lidocaine group and 2.87±2.88 in the articaine group, with no significant difference between the two groups (P=0.3) (Table 2). The means of MBPS values were 4.52±2.55 in the lidocaine group and 3.13±1.86 in the articaine group, indicating significantly better patient behavior in the articaine group (P=0.004) (Table 3). No adverse events were reported in any of the subjects.

**Table 2:** Reported pain values during injection according to the Wong-Baker FACES pain rating scale

**Pain Value**	Articaine BI* (%)	Lidocaine IANB** (%)
**0**	6 (26.1)	6 (26.1)
**2**	9 (39.1)	6 (26.1)
**4**	4 (17.4)	5 (21.7)
**6**	2 (8.7)	3 (13.1)
**8**	0 (0.0)	2 (8.7)
**10**	2 (8.7)	1 (4.3)

*Buccal infiltration **Inferior alveolar nerve block

**Table 3:** Reported pain values during pulp excavation according to the modified behavioral pain scale

**Pain Value**	Articaine BI* (%)	Lidocaine IANB** (%)
**0**	1 (4.3)	2 (8.7)
**1**	0 (0.0)	0 (0.0)
**2**	11 (47.8)	4 (17.4)
**3**	5 (21.7)	2 (8.7)
**4**	1 (4.3)	5 (21.7)
**5**	1 (4.3)	1 (4.3)
**6**	3 (13.0)	2 (8.7)
**7**	0 (0.0)	3 (13.0)
**8**	1 (4.3)	4 (17.4)
**9**	0 (0.0)	0 (0.0)
**10**	0 (0.0)	0 (0.0)

*Buccal nfiltration **Inferior alveolar nerve block

## Discussion

Pain control is mandatory for reducing the fear and anxiety during dental procedures, particularly in children. The aim of this randomized double-blind clinical trial was to evaluate the anesthetic efficacy of 4% articaine BI versus 2% lidocaine IANB for pulpotomy treatment of mandibular primary second molars in 5‒8-year-old children.

In this study, The WBFPRS was used for the assessment of pain during injection. MBPS was used for evaluating children’s behavior during the procedure. In the study by Ram and Amir,^15^ the same scales were used.

Kaufman et al^16^ reported that IANB was significantly more painful than infiltration; however, in this study, there was no significant difference between the two methods.

Nusstein and Beck^17^ and Nakanishi et al^18^ reported that the efficacy of topical anesthesia might be related to the location of the injection site; therefore, applying topical anesthetic gel prior to injection was not advantageous when IANB was used as the delivery route. However, in this study, a 20% benzocaine gel was applied for one minute on the site of injection in both injection techniques.

Similar to the results of Arali and Mytri,^19^ in the current study, 4% articaine BI was more effective than 2% lidocaine IANB in achieving pulpal anesthesia in 5‒8-year-old children, although the treatment and the target teeth were different from the current study. In contrast to the current study, Arrow^20^ reported that IANB was more successful than BI, although the comparison of these studies might not be appropriate due to the difference in the mean age of patients.

Similar to the results of previous studies,^15,20,21^ in this study, the type of local anesthetic solution or the method of administration did not affect the prevalence of post-procedural adverse events.

This was the first study that evaluated the anesthetic efficacy of 4% articaine BI versus 2% lidocaine IANB in mandibular second primary molars. Mandibular infiltration using 4% articaine could be considered as an alternative method to mandibular block anesthesia.

## Conclusions

Based on the results of this study it can be concluded that articaine BI could be a valuable alternative to the lidocaine IANB for pulpotomy of mandibular second primary molars.

## Authors’ contributions

**Inferior alveolar nerve block

The study was planned by SG, ZK and MS. Data collection was carried out by ZK; statistical analyses and interpretation of data were carried out by RB and ZK. The manuscript was prepared by RB and ZK, and edited by SG and MS. All the authors have read and approved the final manuscript for submission.

## Acknowledgments

This study was supported by a grant from Tehran University of Medical Sciences (grant no. T8224/TUMS).

## Funding

This study was supported by Tehran University of Medical Sciences, School of Dentistry.

## Competing interests

The authors declare that they have no competing interests with regards to authorship and/or publications of this paper.

## Ethics approval

The study protocol was approved by the Ethics Committee in Medical Research of Tehran University of Medical Sciences (IR.TUMS.REC.1394.610). The trial was registered in the Iranian registry of Clinical Trials (IRCT2015042321484N2). TWritten informed consent was taken from all the participants.
